# The reduction of nitrogen fertilizer combined with humic acid application improves potato yield by modulating soil microbial community structure in arid and semi-arid regions

**DOI:** 10.3389/fpls.2026.1927454

**Published:** 2026-08-18

**Authors:** Meirong Wang, Peng Liu, Zhihui Shang, Jiawei Guo, Hui Zhou, Jie Liu, Yuanyuan Zhang, Ting Guan, Ru Xu, Xiaohua Shi, Mingshou Fan, Liguo Jia

**Affiliations:** 1 College of Agronomy, Inner Mongolia Agricultural University, Hohhot, China; 2 Ulanqab City Inspection and Testing Center, Ulanqab, China; 3 Institute of Water Resources for Pastoral Area, Ministry of Water Resources, Hohhot, China

**Keywords:** humic acid, nitrogen reduction, PLS-PM, potato, rhizosphere microorganisms, soil nutrients

## Abstract

**Introduction:**

Humic acid is a biostimulant with considerable potential to reduce nitrogen (N) fertilizer input and improve fertilizer use efficiency. However, the mechanisms by which it regulates soil nutrient supply, N utilization, and yield formation in potato remain unclear.

**Methods:**

A single-site, single-season field experiment was conducted in the northern Yinshan Mountains using a randomized block design with a 3 × 3 factorial arrangement of N rates and humic acid applications. Three N levels (300, 270, and 240 kg N ha^−1^) and three humic acid rates (0, 7.5, and 15 L ha^−1^) were established. The effects of reduced N input combined with liquid humic acid application on rhizosphere soil nutrient status, microbial biomass, potential enzyme activities, microbial community composition, N use efficiency, and potato yield were evaluated.

**Results:**

Compared with the conventional N input without humic acid treatment (H0N1), a 20% reduction in N input combined with 15 L ha^−1^ liquid humic acid application (H2N1) increased tuber yield by 13.0%, plant N accumulation and N uptake efficiency by 60.1%, and partial factor productivity of applied N by 13.0%, whereas N utilization efficiency decreased by 29.4%. Humic acid application enhanced rhizosphere soil nutrient availability, microbial biomass carbon and nitrogen, and the potential activities of enzymes involved in carbon and nitrogen transformation. Furthermore, N rate and humic acid application jointly altered bacterial and fungal community composition and promoted treatment-specific enrichment of indicator taxa, with fungal communities showing a stronger response than bacterial communities.

**Discussion:**

Partial least squares path modeling indicated that soil nutrient availability was the strongest direct factor associated with potato yield, whereas humic acid was indirectly associated with yield through changes in microbial community structure, microbial biomass and potential enzyme activities, and soil nutrient availability. Under the conditions of this experiment, reducing N input by 20% combined with 15 L ha^−1^ liquid humic acid application showed potential for reducing N fertilizer use, sustaining potato productivity, and improving rhizosphere soil ecological properties. Multi-year and multi-site field experiments are required to further verify the stability and broader applicability of this strategy.

## 1 Introduction

Potato (*Solanum tuberosum* L.) is one of the world’s most important food crops and plays a vital role in global food security and regional agricultural development (Devaux et al., 2021). Owing to its shallow root system and concentrated nutrient demand during the tuber formation stage, potato is highly sensitive to nitrogen supply, making appropriate nitrogen management a key determinant of achieving both high yield and quality (Koch et al., 2020). The northern Yinshan Mountains in Inner Mongolia constitute one of the major potato-producing regions in China (Bai et al., 2019), characterized by a typical semi-arid agro-ecosystem (Guan et al., 2025; Kong et al., 2024). Although this region benefits from abundant solar radiation and a large diurnal temperature range, it is still constrained by insufficient precipitation, low soil fertility, and limited water and nutrient resources (Chang et al., 2020). In such semi-arid agricultural systems, soil nutrient supply and N use efficiency are often limited, becoming key limiting factors for stable and high potato productivity (Geisseler and Scow, 2014; Kuypers et al., 2018). Although long-term high nitrogen inputs can maintain crop yields, they often reduce nitrogen use efficiency and cause soil nutrient imbalance as well as alter soil microbial processes (Liu et al., 2022). Soil microorganisms, as central drivers of carbon and nitrogen cycling, directly regulate soil fertility and plant nutrient acquisition through shifts in community structure and functional potential (Delgado-Baquerizo et al., 2020; Jansson and Hofmockel, 2020). Therefore, within intensive potato production systems in this region, maintaining soil nutrient supply capacity while improving nitrogen use efficiency under reduced nitrogen input remains a key scientific challenge for sustainable and high-efficiency agriculture.

Humic acid (HA), as an important biostimulant and fertilizer efficiency enhancer, has received increasing attention in recent years (Canellas et al., 2020; Da Silva et al., 2017). Owing to its abundance of functional groups such as carboxyl and phenolic hydroxyl, humic acid can regulate the transformation and mobility of soil ammonium and nitrate through adsorption, complexation, and ion exchange processes, thereby reducing nutrient losses and improving nitrogen availability (Lindsey et al., 2021; Liu et al., 2024). As an exogenous active organic carbon input, humic acid also improves soil structure and enhances microbial metabolic activity, thereby strengthening the coupling of soil carbon and nitrogen cycling processes (Ampong et al., 2022). At the biological level, humic acid can stimulate key hydrolytic enzyme activities, such as urease and invertase, thereby strengthening soil carbon and nitrogen transformation processes while reshape microbial community composition and influence the distribution of microbial taxa associated with nutrient cycling (Benítez et al., 2005). Meanwhile, microbial community stability is considered a key attribute for maintaining the functional sustainability of soil ecosystems and is highly responsive to fertilization disturbances (Griffiths and Philippot, 2013; Guo et al., 2026). Therefore, the coordinated regulation of soil nutrient pools, enzymatic activities, and microbial processes by humic acid may constitute an important biological basis for its nitrogen-saving and yield-enhancing effects.

Previous studies have shown that humic acid can significantly improve crop yield and nitrogen use efficiency under both reduced and conventional nitrogen input conditions by enhancing soil nutrient availability (Kong et al., 2022; Li et al., 2019), and it has exhibited relatively stable yield-increasing and nutrient-use-improving effects across various crops and agroecosystems (Ma et al., 2024; Wang et al., 2020). However, most existing studies have focused on solid humic acid formulations or conventional fertilization systems, and systematic understanding of the synergistic effects of liquid humic acid and nitrogen reduction under fertigation conditions remains limited. In particular, the mechanisms by which this management strategy affects crop productivity by coordinating soil nutrient availability, microbial community responses, and potential enzyme activities remain unclear in semi-arid potato production systems.

## 2 Materials and methods

### 2.1 General situation of the experimental area

**Figure 1:**
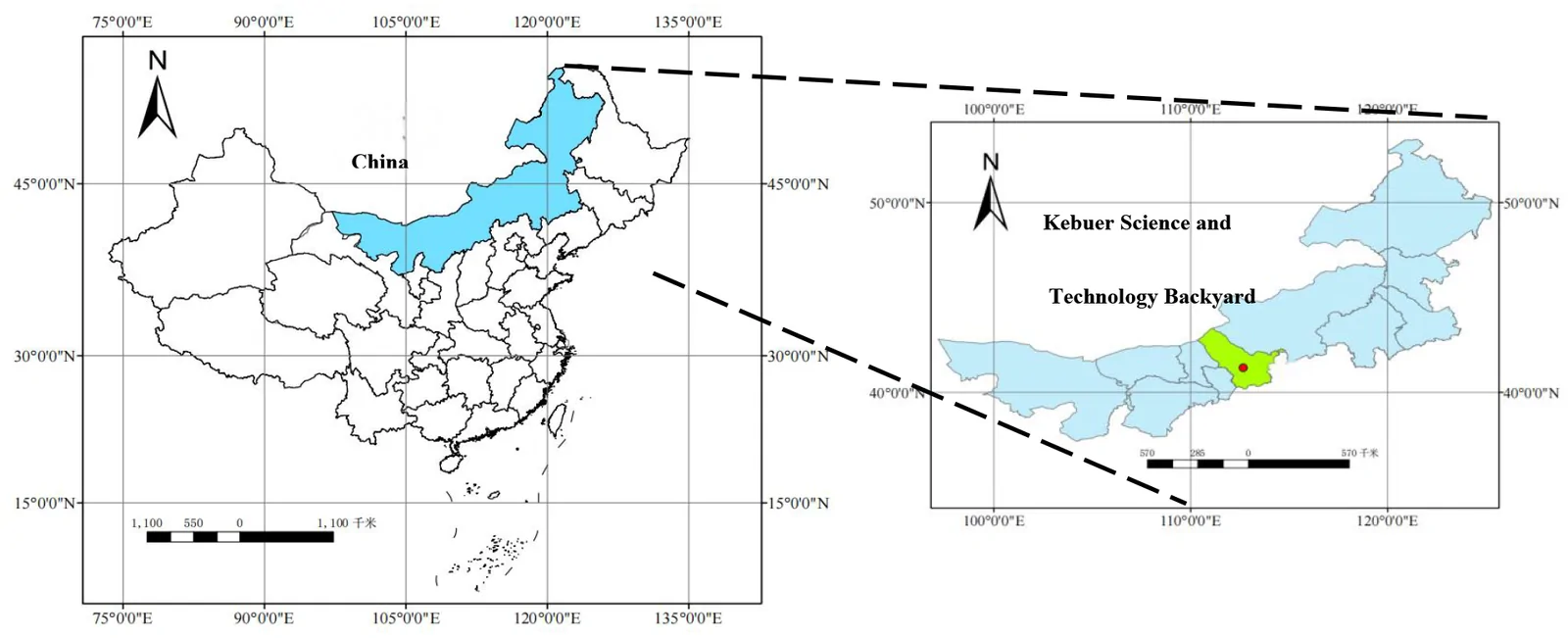
Geographical location of the experimental site.

**Figure 2:**
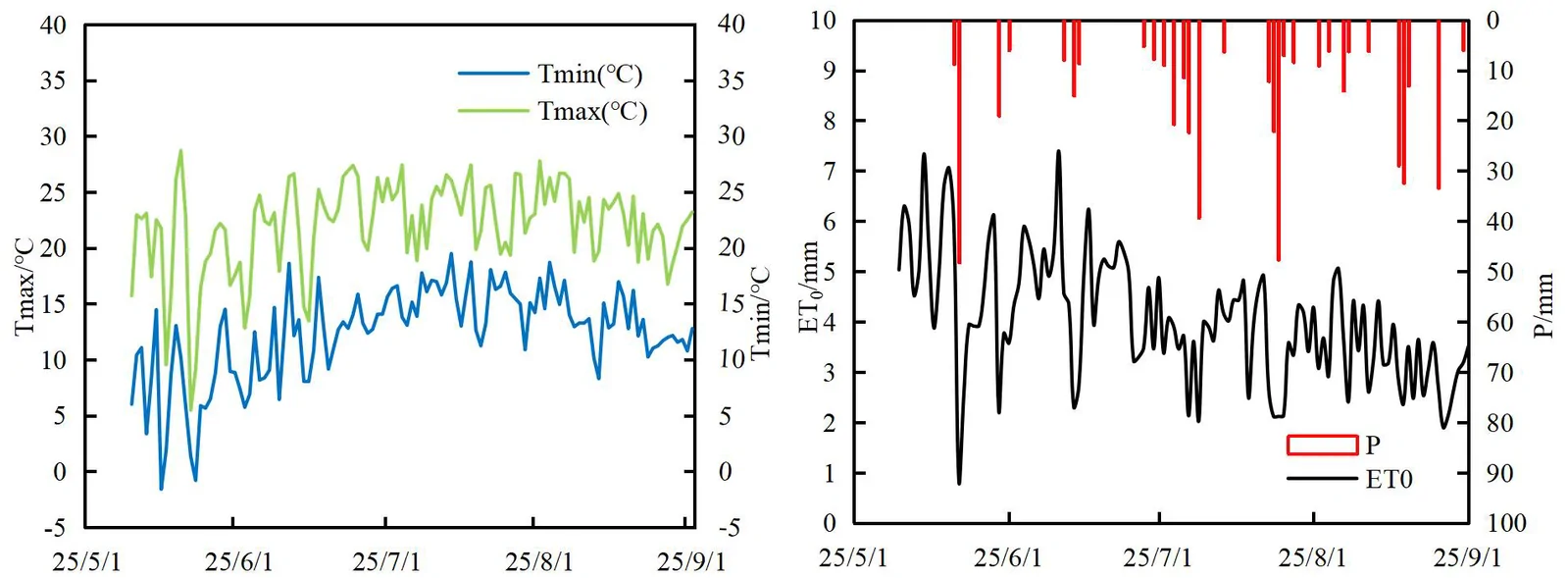
Basic meteorological data of the experimental area.

The experimental site was located in Ke’bo’er, Qahar Right Middle Banner, Ulanqab City, northern Yinshan Mountains, Inner Mongolia Autonomous Region (41°17′59.81″N, 112°33′26.65″E; altitude 1780 m), as shown in **Figure 1**. The region is characterized by a mid-temperate continental climate. The frost-free period is approximately 100 days, with a mean annual temperature of 1.4 °C, a long-term average wind speed of 4.8 m s^−^¹, and a mean annual evaporation of approximately 2000 mm. The long-term average annual precipitation is approximately 300 mm, indicating a typical semi-arid climate. A field microclimate station (HOBO-U30) was installed within the experimental site to automatically record meteorological data. During the potato growing season in 2025, the maximum and minimum air temperatures were 28.7 °C and −1.6 °C, respectively. The reference evapotranspiration (ET_0_) was calculated using the Penman equation based on meteorological data. The effective precipitation (P) was 487.99 mm, and ET_0_ was 464.68 mm during the growing season. The meteorological data are shown in **Figure 2**.

Prior to the experiment in 2025, initial soil samples were collected from the 0–60 cm soil layer. Soil particle size distribution, including sand, silt, and clay fractions at different depths (0–20 cm, 20–40 cm, and 40–60 cm), was determined using a laser particle-size analyzer (Mastersizer 2000, Malvern Instruments, UK). Based on the particle size analysis across soil depths, the arable layer soil in the study area was classified as sandy loam. The initial physicochemical properties of the experimental soil are presented in **Table 1**.

**Table 1:** Physical and chemical properties of the experimental soils in 0–60 cm soil layer.

Soil depth/cm	Particle composition/%			Soil texture	Organic matter/ (g·kg^-1^)
	Clay	Silt	Sand		
0∼20	3.2	38.3	58.5	sandy loam	22.4
>20∼40	8.32	27.98	63.7	sandy loam	21.7
>40∼60	5.52	24.68	69.8	sandy loam	20.8

### 2.2 Experimental design

**Figure 3:**
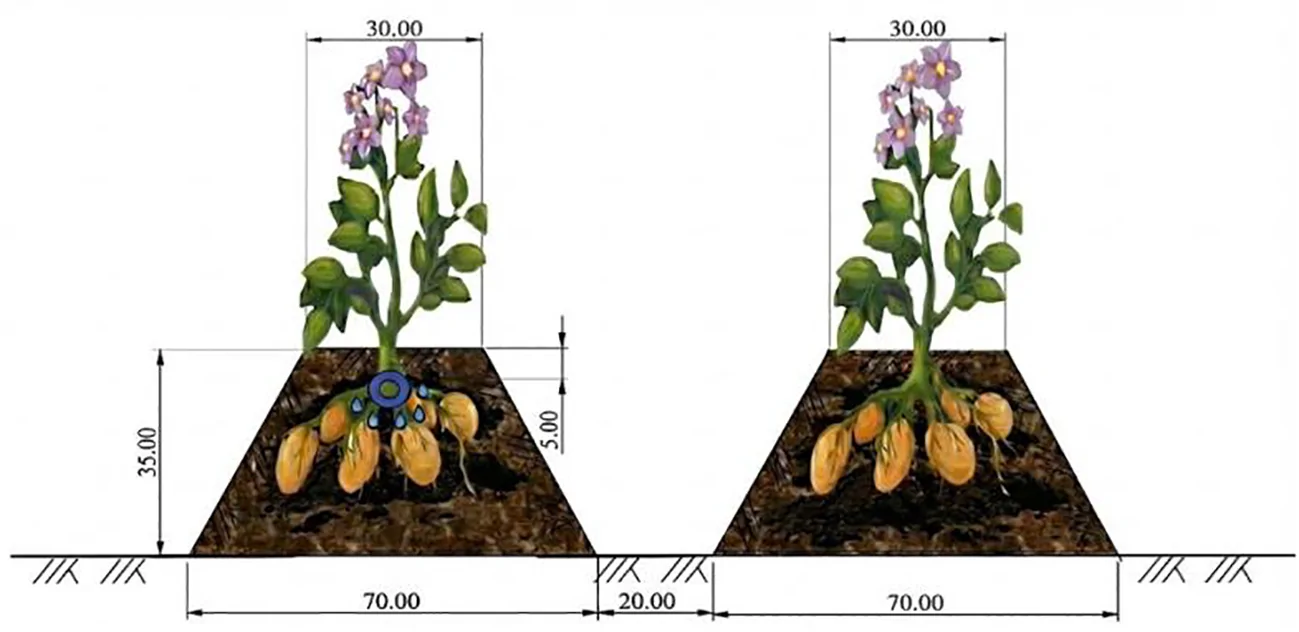
Schematic illustration of potato planting (unit: cm).

The field experiment was conducted from May 12 to September 1, 2025. wheat was the preceding crop. The potato cultivar used in the experiment was ‘Xufeng No. 1’, a widely cultivated drought-tolerant variety in the local region. A local “single-ridge, single-row with drip irrigation under mulch” planting system was adopted. The drip irrigation tape was buried 5 cm below the ridge shoulder center. The drip tape had a wall thickness of 0.18 mm, an emitter spacing of 0.3 m, and a discharge rate of 2.2 L h^−^¹, operating at a pressure of 0.1 MPa. The planting density was 4.63×10^4^ plants ha^−^¹, with a row spacing of 90 cm and a plant spacing of 24 cm. The ridge configuration included a ridge top width of 30 cm, a ridge bottom width of 70 cm, and a ridge height of 35 cm. A schematic diagram of the planting system is shown in **Figure 3**.

The experiment was arranged in a two-factor randomized complete block design based on nitrogen application rate and humic acid dosage. The conventional nitrogen application rate in the local region was set as N3 (300 kg N ha^−^¹), with a 10% and 20% reduction to obtain N2 (270 kg N ha^−^¹) and N1 (240 kg N ha^−^¹), respectively. Under each nitrogen level, three humic acid application rates were established: H2 (15 L ha^−^¹), H1 (7.5 L ha^−^¹), and H0 (0 L ha^−^¹). A total of nine treatment combinations were generated (**Table 2**), each with three replicates, resulting in 27 plots. Each plot covered an area of 140 m² (14 m × 10 m). Urea (46% N) was used as the nitrogen fertilizer. At sowing, 30% of the total nitrogen was applied as a basal fertilizer, while 30% and 40% of the remaining nitrogen were top-dressed at the tuber initiation stage and tuber bulking stage, respectively. The humic acid product used in this study was provided by SCOM Chemical Trade (Shanghai) Co., Ltd. and was derived from weathered lignite sourced from the Gascoigne region of North Dakota, USA. The lignite was micronized using ultra-fine processing equipment and further wet-milled to a particle size of 50 μm to produce a humic acid suspension fertilizer. This liquid formulation is acidic (pH 5–6) and contains humic acid ≥150 g L^−^¹, P_2_O_5_ ≥150 g L^−^¹, and N ≥50 g L^−^¹. Humic acid was applied via drip irrigation in three equal splits: one-third after sowing, one-third at the tuber initiation stage, and one-third at the tuber bulking stage. Phosphorus was supplied as calcium superphosphate (P_2_O_5_, 46%) at a rate of 180 kg ha^−^¹ and applied as a basal fertilizer at sowing. Potassium was supplied as potassium sulfate (K_2_O, 45%) at a rate of 300 kg ha^−^¹ and also applied entirely as basal fertilizer. All other field management practices followed local farmers’ conventional agronomic practices throughout the growing season.

**Table 2:** Experimental design.

Treatment	Basal fertilizer		Tuber initiation stage		Tuber bulking stage		Total	
	N (kg ha^-1^)	HA (L ha^-1^)	N (kg ha^-1^)	HA (L ha^-1^)	N (kg ha^-1^)	HA (L ha^-1^)	N (kg ha^-1^)	HA (L ha^-1^)
H0N3	90	0	120	0	90	0	300	0
H0N2	81	0	108	0	81	0	270	0
H0N1	72	0	96	0	72	0	240	0
H1N3	90	2.5	120	2.5	90	2.5	300	7.5
H1N2	81	2.5	108	2.5	81	2.5	270	7.5
H1N1	72	2.5	96	2.5	72	2.5	240	7.5
H2N3	90	5	120	5	90	5	300	15
H2N2	81	5	108	5	81	5	270	15
H2N1	72	5	96	5	72	5	240	15

### 2.3 Measurement indices and calculation methods

#### 2.3.1 Soil sampling

Rhizosphere soil samples of potato were collected at the tuber bulking stage (45 days after emergence) using a five-point sampling method. Plants with uniform growth and without obvious disease or pest damage were selected. The whole root system was excavated, and loosely attached soil was gently shaken off. Soil tightly adhering to the root surface was collected as rhizosphere soil. The samples were thoroughly mixed, sieved, and placed into sterile bags, and then transported to the laboratory under low-temperature conditions. Each treatment included three replicates. A portion of fresh soil samples was stored at 4 °C for the determination of soil nitrate nitrogen (NO_3_^−^-N), ammonium nitrogen (NH_4_^+^-N), microbial biomass carbon (MBC), microbial biomass nitrogen (MBN), urease activity (UE), and sucrase activity (SC). Air-dried soil samples were used for soil organic carbon (SOC) analysis. Rhizosphere soil tightly adhering to roots was collected under sterile conditions and stored at −80 °C for DNA extraction and high-throughput sequencing analysis.

#### 2.3.2 Soil nutrient determination

Soil organic carbon (SOC) was determined using the Dumas combustion method. Soil samples were combusted at high temperature to convert carbon into CO_2_, which was then quantified using an elemental analyzer (Sato et al., 2014). Soil nitrate nitrogen (NO_3_^−^-N) and ammonium nitrogen (NH_4_^+^-N) were extracted with 2 M KCl solution. After shaking and filtration, the concentrations were determined using a continuous flow analyzer (Fayose et al., 2021).

#### 2.3.3 Soil microbial biomass and enzyme activities

Soil microbial biomass carbon (MBC) and microbial biomass nitrogen (MBN) were measured using the chloroform fumigation–extraction method. Fresh soil samples were divided into fumigated and non-fumigated controls. After fumigation with chloroform, both samples were extracted with K_2_SO_4_ solution, and MBC and MBN were calculated based on the differences in extracted C and N between fumigated and non-fumigated samples (Mori et al., 2021). Soil urease activity (UE) was determined using a urea-based colorimetric method by measuring the released NH_4_^+^-N during incubation (Hu et al., 2021). Soil sucrase activity (SC) was determined using a sucrose-based colorimetric method by quantifying reducing sugars produced from sucrose hydrolysis (Yuan et al., 2017).

#### 2.3.4 Soil DNA extraction and high-throughput sequencing

Total soil DNA was extracted using a commercial soil microbial DNA extraction kit. DNA integrity was checked by agarose gel electrophoresis, and DNA concentration and purity were assessed using a spectrophotometer or fluorometric method. Bacterial 16S rRNA gene fragments and fungal ITS regions were amplified using specific primers. PCR amplification cycles were carefully controlled, and identical conditions were maintained across all samples to minimize amplification bias. PCR products were verified by gel electrophoresis, purified, quantified, and pooled to construct sequencing libraries. Qualified libraries were sequenced on an Illumina high-throughput sequencing platform using paired-end sequencing. Raw reads were processed through quality filtering, merging, chimera removal, and low-quality sequence filtering before downstream analyses of microbial community composition, diversity, and structure (Bolyen et al., 2019).

#### 2.3.5 Potato yield and yield components

At harvest, a 0.9 m×2 m quadrat was sampled in each plot, with three replicates per treatment. Fresh tubers were weighed, and yield (Y, t ha^−^¹) was calculated based on the sampled area. Tubers with individual weight >150 g were classified as marketable tubers, and the ratio of marketable tuber mass to total tuber mass was calculated as marketable yield rate. Average tuber weight per plant was calculated as total tuber mass divided by plant number, and tuber number per plant was calculated as total tuber number divided by plant number (Liu et al., 2023).

#### 2.3.6 Nitrogen use efficiency indices

1. Partial factor productivity of nitrogen (PFP_N_)

Partial factor productivity of nitrogen (PFPN) was used to evaluate the yield return per unit of applied nitrogen. It was calculated according to Equation 1:

(1) $${\text{PFP}}_{\text{N}}=\;1000\text{Y}/{\text{F}}_{\text{N}}$$

Where Y is tuber yield (t ha^−^¹), and FN is nitrogen application rate (kg ha^−^¹). PFPN is expressed as kg kg^−^¹ (Liu et al., 2023).

2. Plant Nitrogen Accumulation (PNA)

Plant nitrogen accumulation (PNA) was defined as the total amount of nitrogen accumulated in the aboveground biomass and tubers per unit land area. It was calculated according to Equation 2:

(2) PNA= SDM × SNC/100 + TDM × TNC/100

Where SDM and TDM are shoot and tuber dry matter (kg ha^−^¹), respectively; SNC and TNC are nitrogen concentrations in shoots and tubers (%); FN is nitrogen application rate (kg ha^−^¹). PNA is expressed as (kg ha^−^¹) (Congreves et al., 2021).

3. Nitrogen uptake efficiency (NUpE)

Nitrogen uptake efficiency was calculated based on total plant nitrogen uptake. It was calculated according to Equation 3:

(3) NUpE=100PNA/FN

Where PNA is total plant nitrogen accumulation (kg ha^−^¹); FN is nitrogen application rate (kg ha^−^¹). NUpE is expressed as % (Congreves et al., 2021).

4. Nitrogen utilization efficiency (NUtE)

Nitrogen utilization efficiency (NUtE) was used to evaluate the ability of crops to convert absorbed nitrogen into yield. It was calculated according to Equation 4:

(4) NUtE=1000Y/UN

Where Y is tuber yield (t ha^−^¹); UN is plant nitrogen uptake (kg ha^−^¹). NUtE is expressed as kg kg^−^¹ (Congreves et al., 2021).

### 2.4 Statistical analysis

Data analysis was performed using SPSS 22.0 for one-way ANOVA followed by LSD multiple comparisons. Two-way ANOVA was used to evaluate the effects of humic acid (H), nitrogen (N), and their interaction (H × N). Microbial alpha and beta diversity were calculated using QIIME2. Non-metric multidimensional scaling (NMDS) and permutational multivariate analysis of variance (PERMANOVA) were used for community structure analysis. Redundancy analysis (RDA) was applied to assess environmental drivers. Linear discriminant analysis effect size (LEfSe) was used to identify differential taxa, and partial least squares path modeling (PLS-PM) was used to construct pathway relationships (Tenenhaus et al., 2005). Figures were generated using R software and Origin.

## 3 Results

### 3.1 Effects of reduced nitrogen combined with humic acid application on rhizosphere soil nutrient dynamics in potato.

As shown in **Table 3**, humic acid application generally increased soil NO_3_^−^-N, NH_4_^+^-N, and SOC contents under the same nitrogen application level. Compared with H0, the H1 treatment changed NO_3_^−^-N, NH_4_^+^-N, and SOC contents by 1.85%–7.61%, −0.44%–3.32%, and 0.67%–1.58%, respectively, whereas the H2 treatment increased these three indicators by 2.54%–8.22%, 5.24%–20.37%, and 2.78%–3.27%, respectively. Compared with H0N1, the H2N1 treatment increased NO_3_^−^-N, NH_4_^+^-N, and SOC contents by 8.0%, 20.4%, and 2.8%, respectively. These results indicate that a higher humic acid application rate exerted a more stable promoting effect on soil nutrient supply, particularly on NH_4_^+^-N accumulation. Two-way ANOVA showed that humic acid and nitrogen application had significant or highly significant effects on NO_3_^−^-N, NH_4_^+^-N, and SOC contents, whereas the H × N interaction had a highly significant effect only on NH_4_^+^-N. This suggests that the combined application of humic acid and nitrogen mainly improved soil nitrogen availability through synergistic regulation of ammonium nitrogen supply.

**Table 3:** Effects of reduced nitrogen application combined with humic acid on soil nutrients.

Treatment	NO_3_--N	NH_4_+-N	SOC
	mg/kg	mg/kg	g/kg
H0N3	17.29 ± 0.52a	9.35 ± 0.28ab	13.36 ± 0.02c
H0N2	16.43 ± 0.49bc	9.04 ± 0.45b	13.44 ± 0.06bc
H0N1	16.00 ± 0.32c	8.10 ± 0.16c	13.30 ± 0.11c
H1N3	17.61 ± 0.53a	9.66 ± 0.29a	13.47 ± 0.13bc
H1N2	17.68 ± 0.35a	9.00 ± 0.18b	13.53 ± 0.19bc
H1N1	17.14 ± 0.17ab	8.32 ± 0.25c	13.51 ± 0.16bc
H2N3	17.73 ± 0.53a	9.84 ± 0.30a	13.79 ± 0.09a
H2N2	17.78 ± 0.53a	9.65 ± 0.29a	13.88 ± 0.19a
H2N1	17.28 ± 0.35a	9.75 ± 0.19a	13.67 ± 0.06ab
HA	**	**	**
N	**	**	ns
HA*N	ns	**	ns

Different lowercase letters within the same column indicate significant differences among treatments at P < 0.05. HA represents humic acid treatment, N represents nitrogen fertilizer treatment, and HA × N represents the interaction between humic acid and nitrogen fertilizer. ns indicates no significant difference; * indicates significance at P < 0.05; ** indicates significance at P < 0.01.

### 3.2 Effects of reduced nitrogen combined with humic acid application on microbial biomass and potential extracellular enzyme activities in potato rhizosphere soil

As shown in **Table 4**, nitrogen reduction and humic acid application affected soil microbial biomass and potential extracellular enzyme activities. At the same humic acid level, reducing the nitrogen application rate from N3 to N1 resulted in changes in MBC ranging from a 15.3% decrease to a 5.5% increase, whereas changes in MBN ranged from a 1.3% decrease to a 5.5% increase. In contrast, the potential activities of UE and SC increased by 8.1%–22.5% and 7.1%–57.3%, respectively. These results indicate that nitrogen reduction did not consistently decrease microbial biomass, whereas the potential activities of enzymes associated with soil carbon and nitrogen transformation generally increased. At the same nitrogen level, relative to H0, H1 increased MBC, MBN, and the potential activities of UE and SC by 8.6%–27.3%, 11.4%–19.1%, 2.1%–12.1%, and 4.7%–25.0%, respectively. The corresponding increases under H2 were 39.1%–73.4%, 17.5%–24.7%, 13.6%–28.9%, and 4.7%–59.5%, respectively, with generally greater increases under H2. In particular, relative to H0N1, H2N1 increased MBC and MBN by 39.1% and 17.5%, respectively, and increased the potential activities of UE and SC by 22.4% and 20.5%, respectively.Two-way ANOVA showed that humic acid significantly affected MBC and SC (P < 0.05) and had a highly significant effect on UE (P < 0.01), but had no significant effect on MBN (P > 0.05). Nitrogen application had highly significant effects on all four variables (P < 0.01), whereas the HA × N interaction significantly affected only SC (P < 0.05).

**Table 4:** Effects of reduced nitrogen combined with humic acid application on microbial biomass and potential extracellular enzyme activities in potato rhizosphere soil.

Treatment	MBC	MBN	UE	SC
	mg/kg	mg/kg	mg/kg	μg/g
H0N3	87.85 ± 9.10d	61.19 ± 6.82c	2.39 ± 0.41c	0.79 ± 0.42c
H0N2	98.34 ± 11.34d	64.18 ± 3.81bc	2.87 ± 0.18bc	1.27 ± 0.17bc
H0N1	92.69 ± 10.40d	64.57 ± 8.15bc	2.72 ± 0.49a	1.12 ± 0.49ab
H1N3	111.80 ± 9.93cd	72.86 ± 6.51ab	2.49 ± 0.36abc	0.89 ± 0.36bc
H1N2	125.07 ± 10.60 bc	71.51 ± 2.97ab	2.93 ± 0.19ab	1.33 ± 0.19bc
H1N1	100.67 ± 11.64d	71.92 ± 3.44ab	3.05 ± 0.18a	1.40 ± 0.21ab
H2N3	152.31 ± 14.14a	73.74 ± 4.81ab	3.08 ± 0.29abc	1.26 ± 0.17bc
H2N2	145.84 ± 10.33ab	80.01 ± 4.79a	3.26 ± 0.32ab	1.33 ± 0.22bc
H2N1	128.96 ± 11.06bc	75.87 ± 5.48a	3.33 ± 0.33a	1.35 ± 0.25a
HA	*	ns	**	*
N	**	**	**	**
HA*N	ns	ns	ns	*

Different lowercase letters within the same column indicate significant differences among treatments at P < 0.05. HA represents humic acid treatment, N represents nitrogen fertilizer treatment, and HA × N represents the interaction between humic acid and nitrogen fertilizer. ns indicates no significant difference; * indicates significance at P < 0.05; ** indicates significance at P < 0.01.

### 3.3 Effects of reduced nitrogen combined with humic acid application on bacterial and fungal community structure in potato rhizosphere soil

#### 3.3.1 Analysis of bacterial and fungal community structure in potato rhizosphere soil

**Figure 4:**
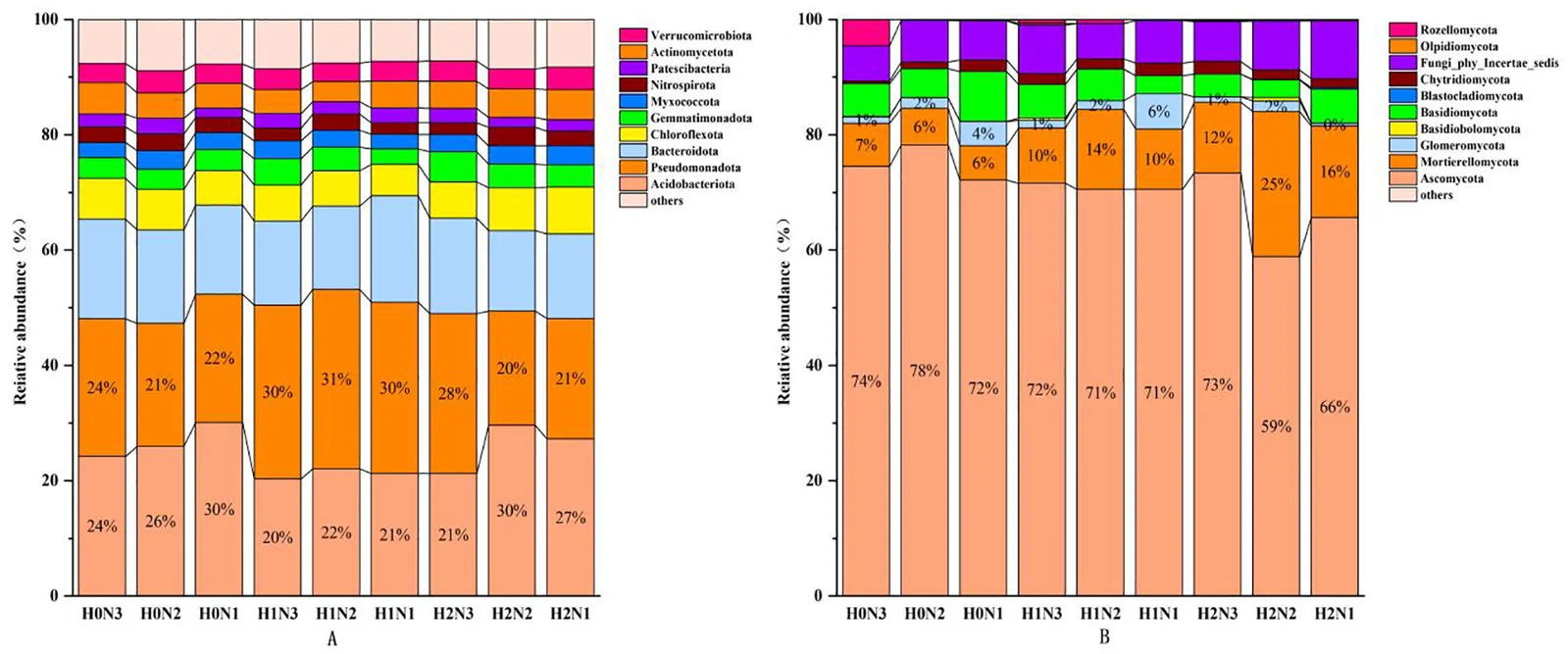
Effects of reduced nitrogen application combined with humic acid on bacterial and fungal community composition in potato rhizosphere soil. **(A)** bacterial phylum-level composition; **(B)** fungal phylum-level composition. Colors indicate different microbial phyla, and bars represent their relative abundances across treatments.

Community composition analysis (**Figure 4**) showed that reduced nitrogen combined with humic acid application exerted varying degrees of influence on the phylum-level composition of soil bacterial and fungal communities. The bacterial community was dominated by *Acidobacteriota, Pseudomonadota*, *Bacteroidota*, *Chloroflexota*, *Gemmatimonadota*, and *Actinobacteriota*. Under the H0 treatment, as nitrogen application decreased from N3 to N1, the relative abundance of *Acidobacteriota* increased from 24% to 30%, whereas that of *Pseudomonadota* decreased from 24% to 22%, indicating that nitrogen reduction alone promoted the enrichment of *Acidobacteriota* while reducing the abundance of *Pseudomonadota*. Following humic acid application, the relative abundance of dominant bacterial phyla showed pronounced treatment-dependent variation. At the N3 level, H1 and H2 treatments increased the relative abundance of *Pseudomonadota* from 24% (H0N3) to 30% and 28%, respectively, while decreasing Acidobacteriota from 24% to 20% and 21%, respectively. At the N2 level, the relative abundance of *Pseudomonadota* increased to 31% under H1N2, whereas *Acidobacteriota* increased to 30% and *Pseudomonadota* decreased to 20% under H2N2. At the N1 level, H1N1 maintained a relatively high abundance of *Pseudomonadota* (30%), whereas H2N1 increased *Acidobacteriota* to 27% and reduced *Pseudomonadota* to 21%. These results suggest that H1 treatment favored the maintenance of higher *Pseudomonadota* abundance, whereas H2 more effectively promoted the enrichment of *Acidobacteriota* under reduced nitrogen conditions. The fungal community was mainly composed of *Ascomycota* and *Mortierellomycota*. Under the H0 treatment, as nitrogen decreased from N3 to N1, the relative abundance of *Ascomycota* decreased slightly from 74% to 72%, while *Mortierellomycota* decreased from 7% to 6%, indicating that nitrogen reduction alone had a limited effect on the dominant fungal taxa. In contrast, humic acid application markedly altered fungal community composition, generally decreasing the relative abundance of *Ascomycota* while increasing that of *Mortierellomycota*. At the N2 level, *Ascomycota* decreased from 78% (H0N2) to 71% (H1N2) and 59% (H2N2), while *Mortierellomycota* increased from 6% to 14% and 25%, respectively. At the N1 level, H2N1 reduced *Ascomycota* from 72% to 66% and increased *Mortierellomycota* from 6% to 16%. Overall, these results indicate that reduced nitrogen combined with humic acid application did not fundamentally alter the dominant microbial phyla, but significantly reshaped soil bacterial and fungal community structures by regulating the relative abundances of dominant taxa.

#### 3.3.2 Alpha diversity of bacterial and fungal communities in potato rhizosphere soil

**Figure 5:**
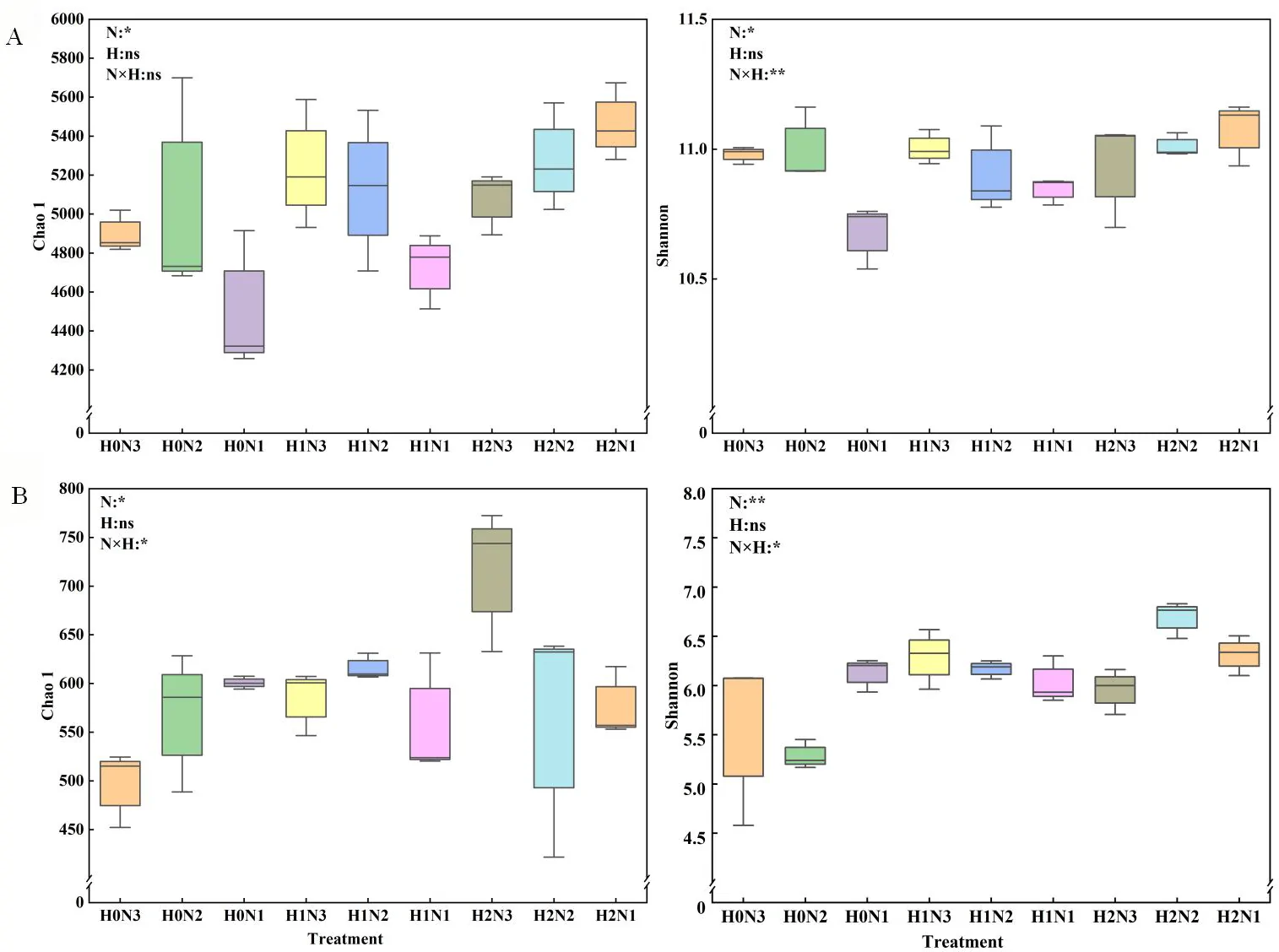
Effects of reduced nitrogen combined with humic acid application on the α-diversity of bacterial and fungal communities in potato rhizosphere soil. **(A)** bacterial community; **(B)** fungal community. Left panels show the Chao1 index, and right panels show the Shannon index. N indicates nitrogen application level, and HA indicates humic acid application level. Boxes represent the interquartile range, the middle line represents the median, and whiskers indicate the range of the data. ns indicates no significant difference; * indicates significance at P < 0.05; ** indicates significance at P < 0.01.

The α-diversity analysis (**Figure 5**) showed that reduced nitrogen combined with humic acid application exerted differential effects on the richness and diversity of soil bacterial and fungal communities. For the bacterial community, under the H0 treatment, decreasing nitrogen application from N3 to N1 reduced the Chao1 index from approximately 4900 to 4300 and the Shannon index from 11.0 to 10.7, indicating that nitrogen reduction alone decreased bacterial richness and diversity. In contrast, humic acid application partially alleviated this negative effect under low nitrogen conditions. In particular, the H2N1 treatment maintained relatively high Chao1 and Shannon indices, suggesting that H2 application could mitigate the adverse effects of nitrogen reduction on bacterial diversity. For the fungal community, under the H0 treatment, decreasing nitrogen application increased the Chao1 index from approximately 500 to 600 and the Shannon index from approximately 5.6 to 6.1, indicating that nitrogen reduction promoted fungal richness and diversity. Humic acid further modified fungal α-diversity patterns. The highest Chao1 value was observed in the H2N3 treatment, whereas the highest Shannon index occurred in the H2N2 treatment, suggesting that H2 application enhanced fungal diversity depending on nitrogen level. Overall, bacterial and fungal communities exhibited opposite responses to nitrogen reduction and humic acid application. Humic acid primarily improved bacterial diversity under low nitrogen conditions and promoted fungal α-diversity under specific nitrogen regimes.

#### 3.3.3 Beta diversity of bacterial and fungal communities in potato rhizosphere soil

**Figure 6:**
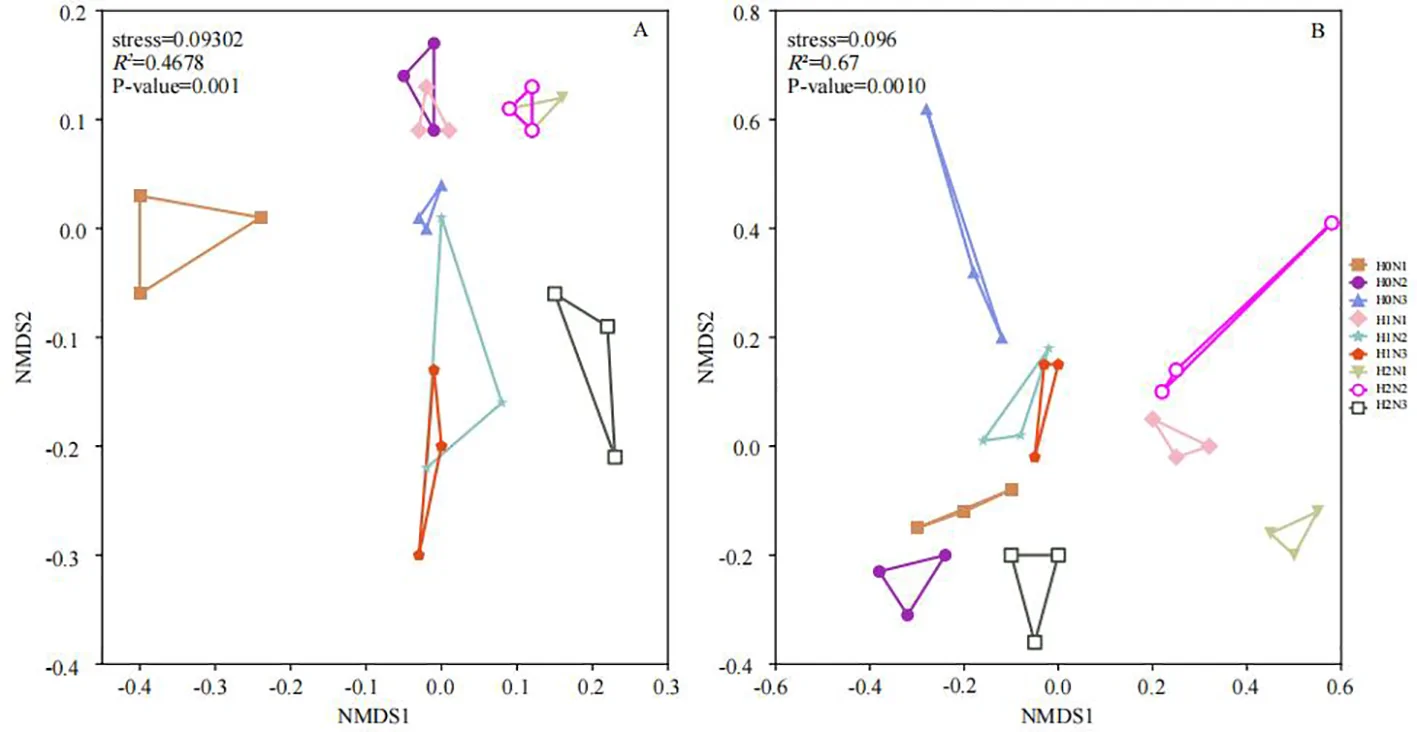
Effects of reduced nitrogen combined with humic acid application on beta diversity of bacterial and fungal communities in potato rhizosphere soil. **(A)** bacterial community; **(B)** fungal community.

The NMDS analysis (**Figure 6**) showed that reduced nitrogen combined with humic acid application significantly altered the bacterial and fungal community structures in the potato rhizosphere soil. For the bacterial community, samples from different treatments were clearly separated along NMDS1 and NMDS2 axes, while samples within the same treatment clustered closely together, indicating distinct differences in bacterial community composition among treatments. The stress value was 0.093, and PERMANOVA analysis showed a significant treatment effect (*R*² = 0.4678, *P* = 0.001), confirming the reliability of the ordination results and the strong influence of treatments on bacterial community structure. Notably, some H2-treated samples were clearly separated from other treatments, suggesting that higher humic acid application exerted a stronger regulatory effect on bacterial community structure. A similar pattern was observed for the fungal community, with clear separation among treatments and relatively tight clustering within each treatment group, indicating distinct fungal community responses. The stress value was 0.096, and PERMANOVA analysis revealed a stronger treatment effect (*R*² = 0.67, *P* = 0.001) compared with the bacterial community. This suggests that fungal communities were more sensitive to combined nitrogen reduction and humic acid application and may play a more responsive role in regulating rhizosphere microbial ecology under these conditions.

#### 3.3.4 Linear discriminant analysis effect size of bacterial and fungal communities in potato rhizosphere soil

**Figure 7:**
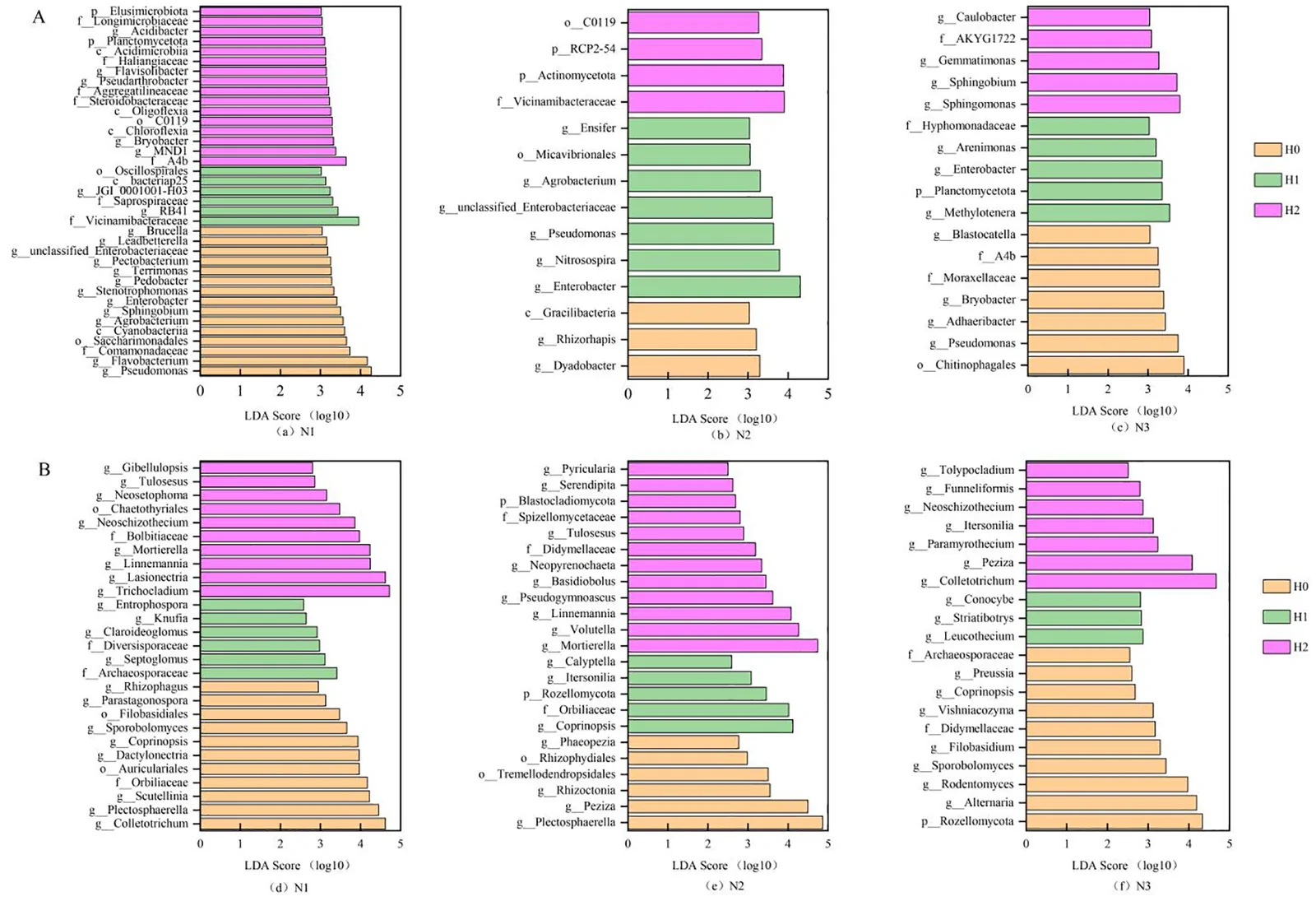
LEfSe analysis of bacterial and fungal communities in potato rhizosphere soil under reduced nitrogen combined with humic acid application. **(A)** bacterial community; **(B)** fungal community.

LEfSe analysis (**Figure 7**) showed that bacterial and fungal communities in the potato rhizosphere exhibited treatment-specific enrichment of indicator taxa under different nitrogen and humic acid regimes. Within the bacterial community, at the N1 level, H0N1, H1N1, and H2N1 were characterized by the enrichment of *Pseudomonas*, RB41, and *Bryobacter*, respectively, indicating that humic acid application altered the composition of bacterial indicator taxa under low-nitrogen conditions. At the N2 level, *Dyadobacter*, *Nitrosospira*, and Actinomycetota were representative of H0N2, H1N2, and H2N2, respectively, while *Pseudomonas* and *Enterobacter* were also enriched under H1N2. At the N3 level, H0N3, H1N3, and H2N3 were characterized by *Pseudomonas*, *Methylotenera*, and *Sphingomonas*, respectively. The fungal community also displayed clear treatment-specific patterns. In particular, at the N1 level, H2N1 was characterized by the enrichment of *Mortierella*, *Linnemannia*, *Trichocladium*, and *Lasionectria*. At the N2 level, differential taxa including *Mortierella* were also detected under H1N2 and H2N2. Overall, nitrogen level and humic acid application jointly altered the distribution of bacterial and fungal indicator taxa in the rhizosphere, reflecting differences in the taxonomic composition of microbial communities under contrasting nutrient-management regimes. However, these findings should not be interpreted as direct evidence of changes in microbial functions or ecological process rates.

#### 3.3.5 Redundancy analysis of environmental factors and microbial community in potato rhizosphere soil

**Figure 8:**
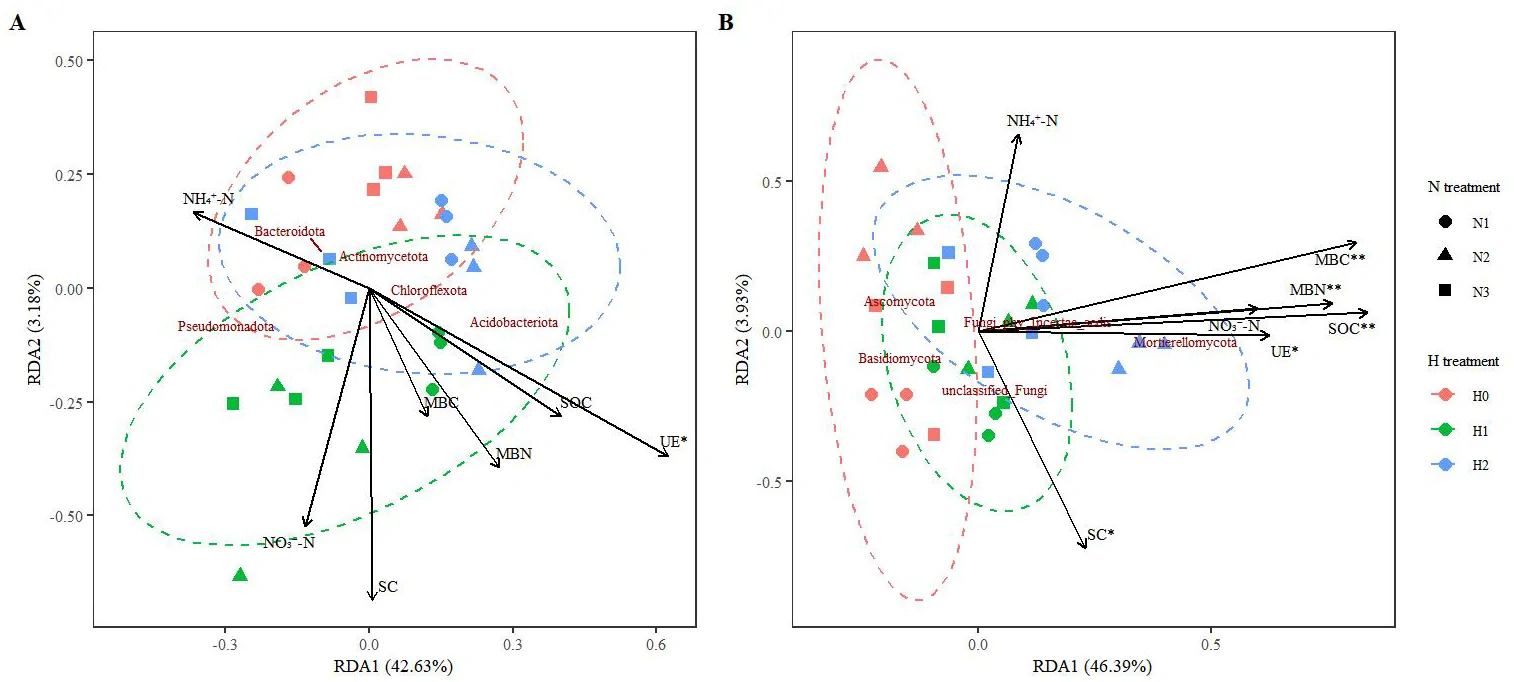
Redundancy analysis (RDA) of environmental factors and microbial communities in potato rhizosphere soil. Different shapes represent nitrogen treatments (N1, N2, and N3), and different colors represent humic acid treatments (H0, H1, and H2). The arrows indicate environmental variables, including soil nutrients, microbial biomass, and enzyme activities. The length of each arrow represents the strength of its relationship with microbial community variation, and the angle between arrows reflects the correlation among variables. Taxa labeled in the ordination plots represent dominant bacterial and fungal groups associated with environmental gradients. **(A)** bacterial community; **(B)** fungal community.

Redundancy analysis (RDA) revealed that soil environmental factors significantly influenced the structure of bacterial and fungal communities in the potato rhizosphere (**Figures 8A, B**). For the bacterial community, RDA1 and RDA2 explained 42.63% and 3.18% of the total variation, respectively, while in the fungal community they explained 46.39% and 3.93%, respectively, indicating that RDA1 was the dominant axis driving microbial community differentiation. Samples under different humic acid treatments showed a clear separation pattern in the ordination space. In particular, H0-treated samples were mainly distributed on the negative side of RDA1, whereas H2-treated samples were mainly located on the positive side of RDA1, suggesting that humic acid application was a key factor shaping rhizosphere microbial community structure. In contrast, separation among nitrogen levels was relatively weak, indicating that nitrogen level had a weaker influence on overall community structure compared with humic acid application. Environmental vectors indicated that SOC, MBC, MBN, and urease activity (UE) were strongly aligned with the positive direction of RDA1, with relatively long arrow lengths, suggesting that soil organic carbon, microbial biomass carbon and nitrogen, and urease activity were key drivers of microbial community variation. In the bacterial community, *Acidobacteriota* and *Chloroflexota* were closely aligned with SOC, MBN, and UE, indicating their potential associations with soil organic carbon accumulation, microbial nitrogen pools, and nitrogen transformation processes. In contrast, *Pseudomonadota* was more closely associated with NH_4_^+^-N, suggesting a stronger response to changes in ammonium availability. In the fungal community, *Mortierellomycota* showed a strong positive association with SOC, MBC, and MBN, whereas *Ascomycota* and *Basidiomycota* were more closely aligned with NH_4_^+^-N, indicating differential responses of fungal taxa to soil carbon and nitrogen resources. Significance testing of environmental factors further showed that SOC, MBC, and MBN had highly significant explanatory effects on fungal community structure, while UE and sucrase activity (SC) were also significant, suggesting that fungal communities were particularly sensitive to changes in soil carbon pools and enzymatic activity. Under the N1 level, H2N1 samples were positioned closer to SOC, MBC, MBN, and UE vectors in both bacterial and fungal RDA ordinations, indicating that high humic acid application under reduced nitrogen conditions strengthened the coupling between microbial communities and soil carbon–nitrogen cycling functions. In the bacterial community, H2N1 was more closely associated with *Acidobacteriota* and *Chloroflexota*, while in the fungal community it showed a closer relationship with *Mortierellomycota*.

### 3.4 Effects of reduced nitrogen combined with humic acid application on potato yield and nitrogen use efficiency

#### 3.4.1 Effects of reduced nitrogen combined with humic acid application on potato yield and yield components

As shown in **Table 5**, under the H0 level, tuber yield in the H0N3 treatment was 58.53 t ha^−^¹, which decreased to 53.17 and 50.36 t ha^−^¹ in the H0N2 and H0N1 treatments, representing reductions of 9.16% and 13.96%, respectively. Under the N1 nitrogen reduction level, tuber yields in the H1N1 and H2N1 treatments were 54.01 and 56.91 t ha^−^¹, respectively, increasing by 7.25% and 13.01% compared with H0N1. Notably, no significant difference was observed between H2N1 and H0N2, indicating that H2 application under reduced nitrogen could partially compensate for yield reduction. Regarding yield components, the highest marketable tuber rate was observed in the H2N3 treatment (60.63%), whereas the lowest value occurred in H0N1 (46.76%). Compared with H0N1, the H2N1 treatment increased marketable tuber rate to 54.24%, representing an increase of 16.00%. Tuber weight per plant ranged from 1.13 to 1.33 kg plant^−^¹, and H2N1 increased this parameter by 13.27% compared with H0N1, approaching the level of H0N2. In contrast, tuber number per plant showed relatively small variation among treatments, with no significant difference between H2N1 and H0N1. Two-way ANOVA indicated that both humic acid application and nitrogen level had highly significant effects on tuber yield and tuber weight per plant, with a significant interaction effect (H × N) on these two parameters. Nitrogen level significantly affected marketable tuber rate, whereas humic acid and the H × N interaction showed no significant effects. None of the factors significantly influenced tuber number per plant. Overall, H2N1 maintained relatively high yield and tuber weight under reduced nitrogen conditions, indicating its potential as a nutrient-management treatment under the conditions of this experiment.

**Table 5:** Effects of reduced nitrogen application combined with humic acid on potato yield and its component factors.

Treatment	Yield(t ha^−^¹)	Marketable tuber rate(%)	Tuber weight per plant(kg plant-1)	Tuber number per plant (per plant-1)
H0N3	58.53 ± 0.57a	59.72 ± 6.49ab	1.32 ± 0.01a	8.13 ± 0.43a
H0N2	53.17 ± 1.79b	52.47 ± 12.64ab	1.2 ± 0.04b	8.96 ± 0.4a
H0N1	50.36 ± 0.41c	46.76 ± 6.75b	1.13 ± 0.01c	8.75 ± 0.66a
H1N3	58.63 ± 0.71a	60.39 ± 3.8ab	1.32 ± 0.02a	8.33 ± 0.97a
H1N2	57.17 ± 1.28a	57.82 ± 0.88ab	1.29 ± 0.03a	8.13 ± 0.66a
H1N1	54.01 ± 1.56b	51.85 ± 10.95ab	1.22 ± 0.04b	9.96 ± 1.12a
H2N3	58.91 ± 0.83a	60.63 ± 3.19a	1.33 ± 0.02a	8.79 ± 1.2a
H2N2	57.07 ± 1.30a	58.53 ± 4.27ab	1.28 ± 0.03a	8.38 ± 1.5a
H2N1	56.91 ± 1.26a	54.24 ± 5.35ab	1.28 ± 0.03a	9.25 ± 1.68a
H	**	ns	**	ns
N	**	*	**	ns
H*N	*	ns	*	ns

Different lowercase letters within the same column indicate significant differences among treatments at P < 0.05. H represents humic acid treatment, N represents nitrogen fertilizer treatment, and H × N represents the interaction between humic acid and nitrogen fertilizer. ns indicates no significant difference; * indicates significance at P < 0.05; ** indicates significance at P < 0.01.

#### 3.4.2 Effects of reduced nitrogen fertilizer application combined with humic acid on nitrogen accumulation and nitrogen use efficiency in potato

**Figure 9:**
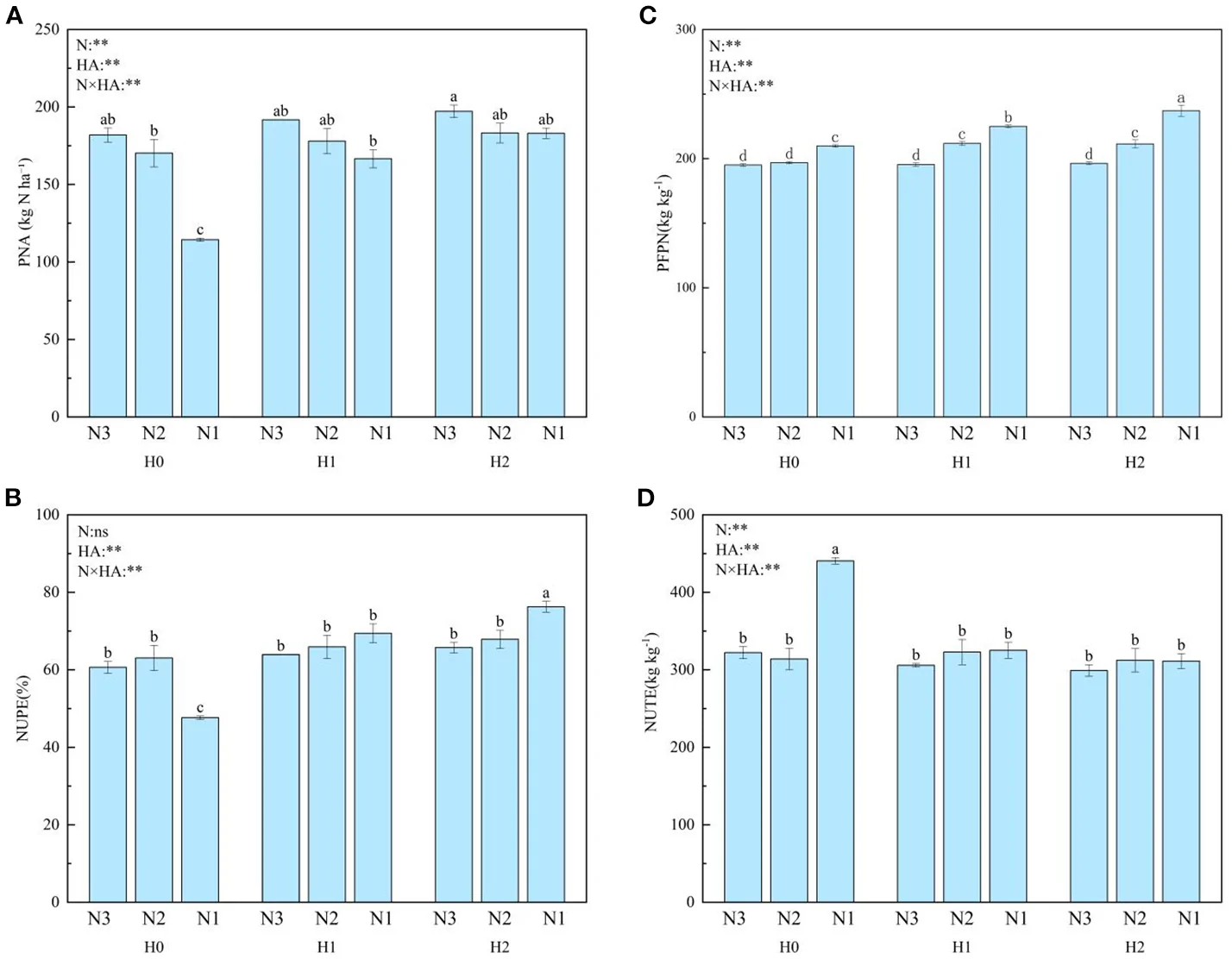
Effects of reduced nitrogen application combined with humic acid application on nitrogen accumulation and nitrogen use efficiency in potato plants. **(A)** Plant nitrogen accumulation; **(B)** nitrogen uptake efficiency (NUpE); **(C)** partial factor productivity of applied nitrogen (PFPN); **(D)** nitrogen utilization efficiency (NUtE). Different lowercase letters indicate significant differences among treatments at *p* < 0.05. N, HA, and N × HA represent the effects of nitrogen application level, humic acid treatment, and their interaction, respectively. ns indicates no significant difference; * indicates significance at *p* < 0.05; ** indicates significance at *p* < 0.01.

As shown in **Figure 9**, at the N1 level, plant nitrogen accumulation (PNA) generally increased with increasing humic acid application rate. Compared with H0N1, PNA under H1N1 and H2N1 increased significantly by 45.7% and 60.1%, respectively. Although PNA under H2N1 was 9.9% higher than that under H1N1, the difference was not significant. Nitrogen uptake efficiency (NUpE) exhibited a similar increasing trend. Compared with H0N1, NUpE increased significantly by 45.7% and 60.1% under H1N1 and H2N1, respectively. Moreover, NUpE under H2N1 was significantly higher than that under H1N1 by 9.9%. The highest partial factor productivity of applied nitrogen (PFP_n_) was observed under H2N1, which was significantly higher than that under H0N1 and H1N1 by 13.0% and 5.4%, respectively. In contrast, nitrogen utilization efficiency (NUtE) was highest under H0N1. Compared with H0N1, NUtE decreased significantly by 26.2% and 29.4% under H1N1 and H2N1, respectively, whereas no significant difference was observed between H1N1 and H2N1. Two-way analysis of variance showed that nitrogen application rate (N), humic acid treatment (HA), and their interaction (N × HA) had highly significant effects on PNA, PFP_n_, and NUtE (*P* < 0.01). Humic acid treatment and the N × HA interaction also had highly significant effects on NUpE (*P* < 0.01), whereas the main effect of nitrogen application rate on NUpE was not significant (*P* > 0.05).

### 3.5 Partial least squares path modeling of potato yield and soil environmental factors

**Figure 10:**
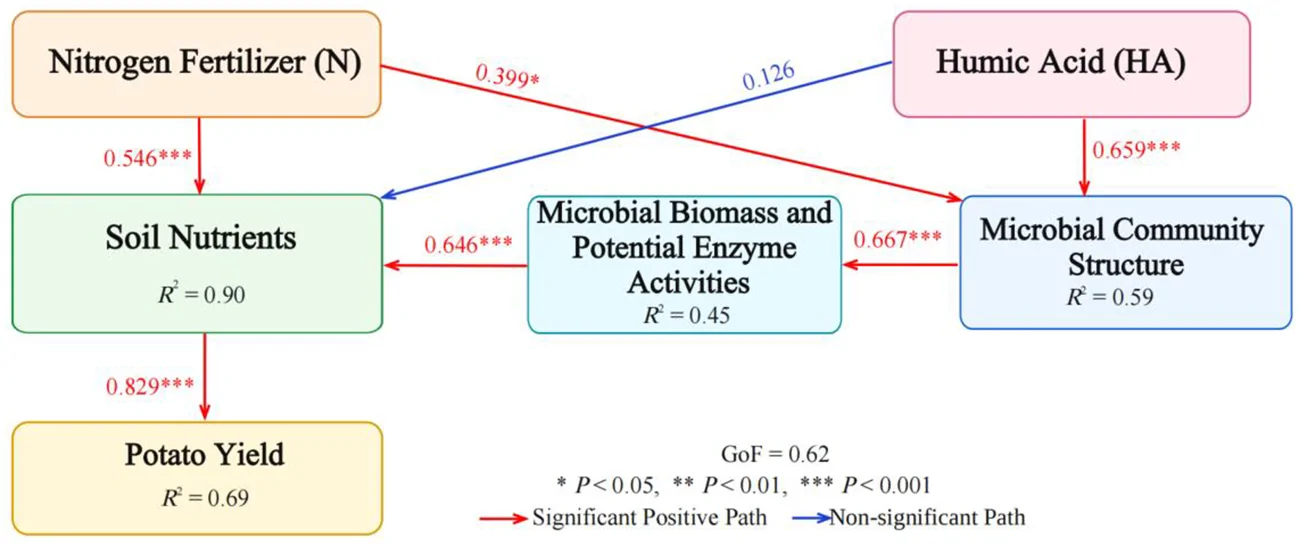
PLS-PM path model illustrating the effects of reduced nitrogen application combined with humic acid on soil microbial biomass and potential enzyme activities, soil nutrient availability, and potato yield. Red lines indicate significant positive effects, whereas blue lines indicate non-significant effects. Numbers adjacent to arrows represent standardized path coefficients. R² represents the explained variance of the model. *P < 0.05, **P < 0.01, ***P < 0.001.

As shown in **Figure 10**, the PLS-PM results indicated that nitrogen fertilizer and humic acid jointly regulated soil nutrient status, microbial biomass and potential enzyme activities, and potato yield through both direct and indirect pathways. The model showed a good explanatory power, with a goodness-of-fit (GoF) value of 0.62. Nitrogen fertilizer had a significant positive direct effect on soil nutrients, with a path coefficient of 0.546, and also significantly affected microbial community structure (path coefficient = 0.399), indicating that nitrogen not only directly improved soil nutrient levels but also participated in nutrient regulation through microbial-mediated pathways. Humic acid showed a stronger positive effect on microbial community structure (path coefficient = 0.659), whereas its direct effect on soil nutrients was relatively weak and non-significant, suggesting that its influence on soil nutrient improvement was mainly mediated through microbial processes. Furthermore, microbial community structure had a significant positive effect on microbial biomass and potential enzyme activities (path coefficient = 0.667), which in turn significantly enhanced soil nutrient availability (path coefficient = 0.646). Soil nutrients exerted the strongest direct effect on potato yield (path coefficient = 0.829), explaining 0.69 of the variance in yield, indicating that soil nutrient availability is the key direct driver of potato yield formation.

## 4 Discussion

### 4.1 Responses of soil nutrient supply and biological activity to reduced nitrogen combined with humic acid application

Under the conditions of this experiment, the results showed that reduced nitrogen combined with humic acid application significantly increased soil NO_3_^−^-N, NH_4_^+^-N, and SOC contents, while also enhancing MBC, MBN, urease activity, and sucrase activity. These results indicate that humic acid can maintain relatively high soil nutrient supply capacity and biological metabolic activity under reduced nitrogen input, highlighting its important role in regulating soil nutrient cycling and microbial processes. The functional mechanisms of humic acid are primarily attributed to its abundant oxygen-containing functional groups, such as carboxyl and phenolic hydroxyl groups, which enhance the adsorption, complexation, and ion exchange capacity of soil systems. These processes can improve the retention of NH_4_^+^-N and NO_3_^−^-N, reduce nitrogen leaching and volatilization losses, and regulate nitrogen release dynamics, thereby enhancing the sustained nitrogen supply capacity of soil (Dong et al., 2009; Rose et al., 2014). In addition, as a stable exogenous source of labile organic carbon, humic acid provides substrates for microbial metabolism, thereby stimulating microbial biomass accumulation and enzyme-mediated reactions, and strengthening soil carbon–nitrogen coupling processes (Ampong et al., 2022; Shade et al., 2012). Previous studies have shown that humic acid improves soil nutrient retention, enhances key enzyme activities, and promotes soil organic matter accumulation (Li et al., 2019; Wang et al., 2020). The present study further confirms, in a semi-arid potato drip-irrigation system, that liquid humic acid under reduced nitrogen conditions can simultaneously enhance soil nutrient availability and microbial biological activity, thereby achieving coordinated regulation of soil carbon and nitrogen cycling processes.

### 4.2 Rhizosphere microbial community restructuring and indicator taxon enrichment in response to reduced nitrogen combined with humic acid application

The rhizosphere microbial community serves as a key ecological interface linking soil nutrient transformation and plant nutrient uptake. This study demonstrated that reduced nitrogen combined with humic acid application significantly altered both bacterial and fungal community structures in the potato rhizosphere. NMDS analysis showed clear separation among treatments, with the most pronounced divergence observed under the 15 L ha^−^¹ humic acid treatment, indicating a strong regulatory effect of humic acid on microbial community structure. RDA further revealed that SOC, MBC, MBN, and urease activity were closely associated with microbial community shifts, suggesting that these shifts were related to changes in rhizosphere carbon and nitrogen availability, microbial biomass, and potential enzyme activities (Geisseler and Scow, 2014; Tian et al., 2017). Previous studies have also shown that humic substances can indirectly drive microbial community restructuring by modifying soil physicochemical properties and rhizosphere microenvironments (Ampong et al., 2022) (Lumactud et al., 2022). LEfSe analysis further indicated that nitrogen application rate and humic acid amendment exerted distinct ecological filtering effects on potato rhizosphere microorganisms, resulting in treatment-specific assemblages of indicator taxa. Notably, under a 20% reduction in nitrogen fertilizer input, the application of 15 L ha^−^¹ humic acid was characterized by the enrichment of several fungal taxa, including Mortierella, Linnemannia, Trichocladium, and Lasionectria. Among these taxa, Mortierella has frequently been associated with organic matter decomposition and nutrient mineralization, and its enrichment may serve as a potential biological indicator of changes in soil fertility (Ozimek and Hanaka, 2021; Sang et al., 2022). Together with the higher microbial biomass and greater potential activities of UE and SC observed under H2N1, these findings suggest that humic acid may have reshaped the rhizosphere microbial community under reduced nitrogen input by altering carbon availability and nutrient conditions, thereby favoring the enrichment of taxa potentially associated with organic matter decomposition and nutrient transformation. Nevertheless, these findings primarily reflect taxonomic shifts in indicator taxa, and their specific ecological functions remain to be validated.

### 4.3 Regulatory pathways of reduced nitrogen combined with humic acid application on potato yield and nitrogen use efficiency

Under the conditions of this experiment, the results showed that reduced nitrogen combined with humic acid application significantly increased potato yield. Under a 20% reduction in nitrogen input, the application of 15 L ha^−^¹ humic acid increased tuber yield by 13.01%, with no significant difference from the conventional nitrogen treatment, indicating that humic acid partially compensated for the yield reduction caused by reduced nitrogen input. Under a 20% reduction in nitrogen input, humic acid may have increased PNA and NUpE by promoting root growth and enhancing plasma membrane H^+^-ATPase activity and nitrate transport capacity (Canellas et al., 2002; Tavares et al., 2017). Because the nitrogen application rate was identical among these treatments, the increase in tuber yield resulted in a higher PFP_n_. However, the proportional increase in PNA was substantially greater than that in tuber yield, leading to a decline in NUtE. This result indicates that the additionally absorbed nitrogen was not converted into tuber yield to the same extent. This response may be attributable to greater nitrogen allocation to aboveground vegetative organs or restricted nitrogen translocation to the tubers, reflecting a trade-off between enhanced nitrogen acquisition and reduced post-uptake nitrogen utilization efficiency. Previous studies in sweet potato and other crop systems have also reported that humic acid significantly improves yield and nitrogen use efficiency (Chen et al., 2017). Previous studies have further demonstrated that humic acid can enhance crop productivity by improving soil properties and reshaping microbial community structure, with SOC and MBN identified as key driving factors (Ni et al., 2024). The PLS-PM analysis further revealed that humic acid indirectly affected yield formation mainly through a pathway involving “microbial community structure–microbial biomass and potential enzyme activities–soil nutrients–yield” (Kuypers et al., 2018).

Compared with previous studies, this study focused on a liquid humic acid application system under semi-arid potato drip irrigation and integrated soil nutrient availability, potential enzyme activities, microbial community composition, indicator taxa, and nitrogen use efficiency. Overall, the results suggest that the yield response to reduced nitrogen input combined with humic acid application may be associated with improved rhizosphere carbon and nitrogen availability, increased microbial biomass and potential enzyme activities, and shifts in microbial community composition and indicator taxa. These changes may contribute to soil nitrogen supply, plant nitrogen uptake, and potato yield under the conditions of this study.

### 4.4 Study limitations

Several limitations of the present study should be acknowledged. This study was conducted at a single site during a single growing season in a major potato-producing area in the northern foothills of the Yinshan Mountains, and effective precipitation during the 2025 growing season was relatively high. In this region, supplemental irrigation is required for potato production in both normal and dry years, while differences among moisture regimes mainly alter the relative contributions of rainfall and irrigation to crop water supply. Insufficient water supply during critical growth stages may affect the response to reduced nitrogen input combined with humic acid application by restricting soil nitrogen transformation, root nutrient uptake, and microbial activity. Therefore, the findings of this study require further validation through multi-year and multi-site experiments conducted under contrasting water conditions.

In addition, nitrate leaching, ammonia volatilization, nitrous oxide emissions, and other pathways of nitrogen loss were not measured in this study; consequently, the environmental benefits of this management practice could not be quantitatively assessed. Future studies should integrate nitrogen balance in the soil–crop system with measurements of gaseous nitrogen emissions and nitrate leaching to comprehensively evaluate its effects on agricultural nitrogen losses and associated environmental risks.

## 5 Conclusions

A 20% reduction in nitrogen input combined with the application of 15 L ha^−^¹ liquid humic acid increased potato yield, primarily by enhancing plant nitrogen uptake and accumulation. Humic acid application also increased rhizosphere soil nutrient availability, microbial biomass carbon and nitrogen, and the potential activities of enzymes involved in carbon and nitrogen transformation. In addition, it altered bacterial and fungal community composition and was accompanied by treatment-specific enrichment of indicator taxa, with fungal communities showing a more pronounced response than bacterial communities. PLS-PM analysis further indicated that soil nutrient availability was the strongest direct factor associated with potato yield, whereas humic acid was indirectly associated with yield through sequential changes in microbial community structure, microbial biomass and potential enzyme activities, and soil nutrient availability. Overall, applying 15 L ha^−^¹ liquid humic acid with a 20% reduction in nitrogen input showed potential as a nutrient-management practice for reducing nitrogen fertilizer use, sustaining potato productivity, and improving the rhizosphere soil environment under the conditions examined in this study. Nevertheless, multi-year and multi-site field experiments are required to assess the stability and broader applicability of this practice.

## Data Availability

The datasets used in this study have been deposited in the NCBI Sequence Read Archive (SRA) database under the accession numbers PRJNA1510535 and PRJNA1510563. Further inquiries can be directed to the corresponding author.

## References

[B1] Ampong K. Thilakaranthna M. S. Gorim L. Y. (2022). Understanding the role of humic acids on crop performance and soil health. Front. Agron. 4. doi: 10.3389/fagro.2022.848621

[B2] Bai Y. Han S. Gao Y. Zhang W. Fan G. Qiu C. . (2019). Genetic diversity of Potato virus Y in potato production areas in Northeast China. Plant Dis. 103, 289–297. doi: 10.1094/PDIS-04-18-0687-RE 30501466

[B3] Benítez E. Sainz H. Nogales R. (2005). Hydrolytic enzyme activities of extracted humic substances during the vermicomposting of a lignocellulosic olive waste. Bioresour. Technol. 96, 785–790. doi: 10.1016/j.biortech.2004.08.010 15607191

[B4] Bolyen E. Rideout J. R. Dillon M. R. Bokulich N. A. Abnet C. C. Al-Ghalith G. A. . (2019). Reproducible, interactive, scalable and extensible microbiome data science using QIIME 2. Nat. Biotechnol. 37, 852–857. doi: 10.1038/s41587-019-0209-9 31341288 PMC7015180

[B5] Canellas L. P. Canellas N. O. A. da S. Irineu L. E. S. Olivares F. L. Piccolo A. (2020). Plant chemical priming by humic acids. Chem. Biol. Technol. Agric. 7, 11. doi: 10.1186/s40538-020-00178-4 38164791

[B6] Canellas L. P. Olivares F. L. Okorokova-Façanha A. L. Façanha A. R. (2002). Humic acids isolated from earthworm compost enhance root elongation, lateral root emergence, and plasma membrane H^+^-ATPase activity in maize roots. Plant Physiol. 130, 1951–1957. doi: 10.1104/pp.007088 12481077 PMC166705

[B7] Chang L. Han F. Chai S. Cheng H. Yang D. Chen Y. (2020). Straw strip mulching affects soil moisture and temperature for potato yield in semiarid regions. Agron. J. 112, 1126–1139. doi: 10.1002/agj2.20103 41531421

[B8] Chen X. Kou M. Tang Z. Zhang A. Li H. (2017). The use of humic acid urea fertilizer for increasing yield and utilization of nitrogen in sweet potato. Plant Soil Environ. 63, 201–206. doi: 10.17221/24/2017-PSE 42533291

[B9] Congreves K. A. Otchere O. Ferland D. Farzadfar S. Williams S. Arcand M. M. (2021). Nitrogen use efficiency definitions of today and tomorrow. Front. Plant Sci. 12. doi: 10.3389/fpls.2021.637108 34177975 PMC8220819

[B10] Da Silva S. F. Olivares F. L. Canellas L. P. (2017). The biostimulant manufactured using diazotrophic endophytic bacteria and humates is effective to increase sugarcane yield. Chem. Biol. Technol. Agric. 4, 37. doi: 10.1186/s40538-017-0106-8 38164791

[B11] Delgado-Baquerizo M. Reich P. B. Trivedi C. Eldridge D. J. Abades S. Alfaro F. D. . (2020). Multiple elements of soil biodiversity drive ecosystem functions across biomes. Nat. Ecol. Evol. 4, 210–220. doi: 10.1038/s41559-019-1084-y 32015427

[B12] Devaux A. Goffart J.-P. Kromann P. Andrade-Piedra J. Polar V. Hareau G. (2021). The potato of the future: opportunities and challenges in sustainable agri-food systems. Potato Res. 64, 681–720. doi: 10.1007/s11540-021-09501-4 34334803 PMC8302968

[B13] Dong L. Cordova-Kreylos A. L. Yang J. Yuan H. Scow K. M. (2009). Humic acids buffer the effects of urea on soil ammonia oxidizers and potential nitrification. Soil Biol. Biochem. 41, 1612–1621. doi: 10.1016/j.soilbio.2009.04.023 22267875 PMC3260537

[B14] Fayose T. Thomas E. Radu T. Dillingham P. Ullah S. Radu A. (2021). Concurrent measurement of nitrate and ammonium in water and soil samples using ion-selective electrodes: tackling sensitivity and precision issues. Anal. Sci. Adv. 2, 279–288. doi: 10.1002/ansa.202000124 38716159 PMC10989628

[B15] Geisseler D. Scow K. M. (2014). Long-term effects of mineral fertilizers on soil microorganisms—a review. Soil Biol. Biochem. 75, 54–63. doi: 10.1016/j.soilbio.2014.03.023 38826717

[B16] Griffiths B. S. Philippot L. (2013). Insights into the resistance and resilience of the soil microbial community. FEMS Microbiol. Rev. 37, 112–129. doi: 10.1111/j.1574-6976.2012.00343.x 22568555

[B17] Guan T. Liu P. Jia L. Wu L. Qin Y. Shi X. . (2025). Shallow burial of drip irrigation tape improves the water use efficiency of potatoes in semi-arid areas. Sci. Rep. 15, 5263. doi: 10.1038/s41598-025-89295-4 39956826 PMC11830772

[B18] Guo Z. Liu C. Jin L. Hua K. Zhan L. Wang D. . (2026). Soil microbial community stability is regulated by soil bulk density and pH under contrasting long-term fertilization. Eur. J. Soil Sci. 77, e70267. doi: 10.1111/ejss.70267 40046247

[B19] Hu X. Liu X. Qiao L. Zhang S. Su K. Qiu Z. . (2021). Study on the spatial distribution of ureolytic microorganisms in farmland soil around tailings with different heavy metal pollution. Sci. Total Environ. 775, 144946. doi: 10.1016/j.scitotenv.2021.144946 33618300

[B20] Jansson J. K. Hofmockel K. S. (2020). Soil microbiomes and climate change. Nat. Rev. Microbiol. 18, 35–46. doi: 10.1038/s41579-019-0265-7 31586158

[B21] Koch M. Naumann M. Pawelzik E. Gransee A. Thiel H. (2020). The importance of nutrient management for potato production Part I: plant nutrition and yield. Potato Res. 63, 97–119. doi: 10.1007/s11540-019-09431-2 30311153

[B22] Kong S. Qin Y. Shi X. Yu J. Jia L. Chen Y. . (2024). Establishing a critical phosphorus dilution curve for potato in semi-arid regions based on a Bayesian analysis. Front. Plant Sci. 15, 1458741. doi: 10.3389/fpls.2024.1458741 39354945 PMC11442389

[B23] Kong B. Wu Q. Li Y. Zhu T. Ming Y. Li C. . (2022). The application of humic acid urea improves nitrogen use efficiency and crop yield by reducing the nitrogen loss compared with urea. Agriculture 12. doi: 10.3390/agriculture12121996 30654563

[B24] Kuypers M. M. M. Marchant H. K. Kartal B. (2018). The microbial nitrogen-cycling network. Nat. Rev. Microbiol. 16, 263–276. doi: 10.1038/nrmicro.2018.9 29398704

[B25] Li Y. Fang F. Wei J. Wu X. Cui R. Li G. . (2019). Humic acid fertilizer improved soil properties and soil microbial diversity of continuous cropping peanut: a three-year experiment. Sci. Rep. 9, 12014. doi: 10.1038/s41598-019-48620-4 31427666 PMC6700118

[B26] Lindsey A. J. Thoms A. W. McDaniel M. D. Christians N. E. (2021). Plant-available soil nitrogen fluxes and turfgrass quality of Kentucky bluegrass fertilized with humic substances. Crop Sci. 61, 4416–4424. doi: 10.1002/csc2.20592 41531421

[B27] Liu K. Meng M. Zhang T. Chen Y. Yuan H. Su T. (2023). Quantitative analysis of source–sink relationships in two potato varieties under different nitrogen application rates. Agronomy 13, 1083. doi: 10.3390/agronomy13041083 30654563

[B28] Liu Q. Wu K. Song W. Zhong N. Wu Y. Fu X. (2022). Improving crop nitrogen use efficiency toward sustainable green revolution. Annu. Rev. Plant Biol. 73, 523–551. doi: 10.1146/annurev-arplant-070121-015752 35595292

[B29] Liu M. Xu M. Yuan L. Zhang S. Li Y. Zhao B. (2024). Characteristics of N transformation of humic acid urea in different circle layers of the fertisphere: a simulated experiment. Agronomy 14, 223. doi: 10.3390/agronomy14010223 30654563

[B30] Lumactud R. A. Gorim L. Y. Thilakarathna M. S. (2022). Impacts of humic-based products on the microbial community structure and functions toward sustainable agriculture. Front. Sustain. Food. Syst. 6. doi: 10.3389/fsufs.2022.977121

[B31] Ma Y. Cheng X. Zhang Y. (2024). The impact of humic acid fertilizers on crop yield and nitrogen use efficiency: a meta-analysis. Agronomy 14, 2763. doi: 10.3390/agronomy14122763 30654563

[B32] Mori T. Wang S. Wang C. Mo J. Zhang W. (2021). Is microbial biomass measurement by the chloroform fumigation extraction method biased by experimental addition of N and P? iForest 14, 408–412. doi: 10.3832/ifor3374-014

[B33] Ni H. Zhao J. Yang Z. (2024). Effects of compound fertilizer decrement and water-soluble humic acid fertilizer application on soil properties, bacterial community structure, and shoot yield in Lei bamboo (Phyllostachys praecox) plantations in subtropical China. Forests 15, 400. doi: 10.3390/f15030400 30654563

[B34] Ozimek E. Hanaka A. (2021). Mortierella species as the plant growth-promoting fungi present in the agricultural soils. Agriculture 11, 7. doi: 10.3390/agriculture11010007 30654563

[B35] Rose M. T. Patti A. F. Little K. R. Brown A. L. Jackson W. R. Cavagnaro T. R. (2014). A meta-analysis and review of plant-growth response to humic substances: practical implications for agriculture. Adv. Agron. 124, 37–89. doi: 10.1016/B978-0-12-800138-7.00002-4 38826717

[B36] Sang Y. Jin L. Zhu R. Yu X.-Y. Hu S. Wang B.-T. . (2022). Phosphorus-solubilizing capacity of Mortierella species isolated from rhizosphere soil of a poplar plantation. Microorganisms 10, 2361. doi: 10.3390/microorganisms10122361 36557615 PMC9785298

[B37] Sato J. H. de Figueiredo C. C. Marchão R. L. Madari B. E. Benedito L. E. C. Busato J. G. . (2014). Methods of soil organic carbon determination in Brazilian savannah soils. Sci. Agric. 71, 302–308. doi: 10.1590/0103-9016-2013-0306 41099703

[B38] Shade A. Peter H. Allison S. D. Baho D. L. Berga M. Bürgmann H. . (2012). Fundamentals of microbial community resistance and resilience. Front. Microbiol. 3. doi: 10.3389/fmicb.2012.00417 23267351 PMC3525951

[B39] Tavares O. C. H. Santos L. A. Ferreira L. M. Sperandio M. V. L. da Rocha J. G. García A. C. . (2017). Humic acid differentially improves nitrate kinetics under low- and high-affinity systems and alters the expression of plasma membrane H^+^-ATPases and nitrate transporters in rice. Ann. Appl. Biol. 170, 89–103. doi: 10.1111/aab.12317 40046247

[B40] Tenenhaus M. Esposito Vinzi V. Chatelin Y.-M. Lauro C. (2005). PLS path modeling. Comput. Stat. Data Anal. 48, 159–205. doi: 10.1016/j.csda.2004.03.005 38826717

[B41] Tian J. Lou Y. Gao Y. Fang H. Liu S. Xu M. . (2017). Response of soil organic matter fractions and composition of microbial community to long-term organic and mineral fertilization. Biol. Fertil. Soils 53, 523–532. doi: 10.1007/s00374-017-1189-x 30311153

[B42] Wang H. Wu J. Li G. Yan L. (2020). Changes in soil carbon fractions and enzyme activities under different vegetation types of the northern Loess Plateau. Ecol. Evol. 10, 12211–12223. doi: 10.1002/ece3.6852 33209282 PMC7663064

[B43] Yuan Z. Liu H. Han J. Sun J. Wu X. Yao J. (2017). Monitoring soil microbial activities in different cropping systems using combined methods. Pedosphere 27, 138–146. doi: 10.1016/S1002-0160(15)60100-X

